# Hepatitis B virus core antigen as a carrier for *Chlamydia trachomatis* MOMP multi-epitope peptide enhances protection against genital chlamydial infection

**DOI:** 10.18632/oncotarget.6533

**Published:** 2015-12-09

**Authors:** Pengfei Jiang, Wangqi Du, Yirong Xiong, Yan Lv, Juan Feng, Shanli Zhu, Xiangyang Xue, Shao Chen, Lifang Zhang

**Affiliations:** ^1^ Institute of Molecular Virology and Immunology, Department of Microbiology and Immunology, School of Basic Medical Sciences, Wenzhou Medical University, Wenzhou, Zhejiang, P. R. China

**Keywords:** HBcAg, chlamydia trachomatis, major outer membrane protein, multi-epitope peptide, vaccine, Immunology and Microbiology Section, Immune response, Immunity

## Abstract

*Chlamydia trachomatis* (*Ct*) is the leading cause of sexually transmitted diseases worldwide. There is no safe and effective vaccine to control the spread of *Ct*. In development of *Ct* vaccine, selection of appropriate candidate antigens and an effective delivery system may be the main challenges. Multi-epitope of major outer membrane protein (MOMPm) is the most suitable candidate for a *Ct* vaccine, while hepatitis B virus core antigen (HBcAg) has unique advantages as vaccine delivery system. Therefore, in this study, we evaluated the immunogenicity and protective immune response of a novel candidate vaccine in a murine model of chlamydial genital infection. This candidate vaccine comprises MOMPm peptide delivered with HBcAg. Our results of *Ct*-specific serum IgG and secretory IgA assay, cytokine assay, and cytotoxic T-lymphocyte assay revealed that immunogenicity of the candidate vaccine was much better than that of the corresponding synthetic MOMPm peptide. Furthermore, the protective effect of the candidate vaccine was also shown much better than that of the synthetic peptide by calculating the isolation of Chlamydia from vaginal swabs and histopathological analysis. Taken together, our results indicate that HBcAg carrying *Ct* MOMPm could be an effective immune prophylactic for chlamydial infection.

## INTRODUCTION

*Chlamydia trachomatis* (*Ct*), an obligate intracellular parasite, is the main cause of preventable blindness and the leading cause of bacterial sexually transmitted diseases worldwide [1]. The World Health Organization estimates that almost 100 million chlamydial cases occur each year [2]. Although very effective antimicrobial therapy is available, chlamydial infection can recur easily. Therefore, the best and most economical solution to control or eradicate the spread of *Ct* is to develop safe and effective vaccines. Although considerable effort has been expended toward this goal, an effective vaccine has not yet been developed.

Current challenges in the development of *Ct* vaccine include selection of appropriate candidate antigens and an effective delivery system [3]. Therefore, it is necessary to identify protective *Ct* antigens or epitopes in animal models. The major outer membrane protein (MOMP), one of the highly conserved surface associated proteins among the different serotypes of *Ct* [4, 5], may be the most suitable candidate for a *Ct* vaccine because it contains both T- and B-cell epitopes that can induce specific anti-*Ct* immune responses. However, it is difficult to produce recombinant MOMP in a native form on a scale large enough to be commercially viable [6]. At present, multi-epitopes of MOMP (MOMPm), T- and B-cell epitope-rich clusters, were selected to design *Ct* vaccines.

It is reported that synthetic epitope peptides are weak immunogens and may limit the potential protective immune responses [7]. To enhance the immunogenicity of multi-epitope, it can be fused to an innocuous but highly antigenic protein, such as the Hepatitis B virus core antigen (HBcAg). HBcAg can self-assemble into virus-like particle (VLP) and has been generally used as vaccine delivery system [8]. As reported in previous studies, through genetic fusion, the HBcAg protein lends itself to accommodate foreign epitopes in three ways: antigens can be linked to the N-terminus or C-terminus of HBcAg, or inserted into the major immunodominant region (MIR) of HBcAg [9]. In consideration of the fact that *Ct* serovars D and E were the most predominant serovars prevalent worldwide [10-12], in this study, we assessed the HBcAg platform as a delivery system for MOMPm of *Ct* serovar E. Our results showed that the fusion of MOMPm to HBcAg induced specific protective immune response against genital chlamydial infection and that different ways of *Ct* MOMPm fused with HBcAg could induce different levels of protective immune response against genital chlamydial infection.

## RESULTS

### Obtainment of *Ct* MOMPm and formation of HBcAg/MOMPm chimeric VLPs

**Figure 1:**
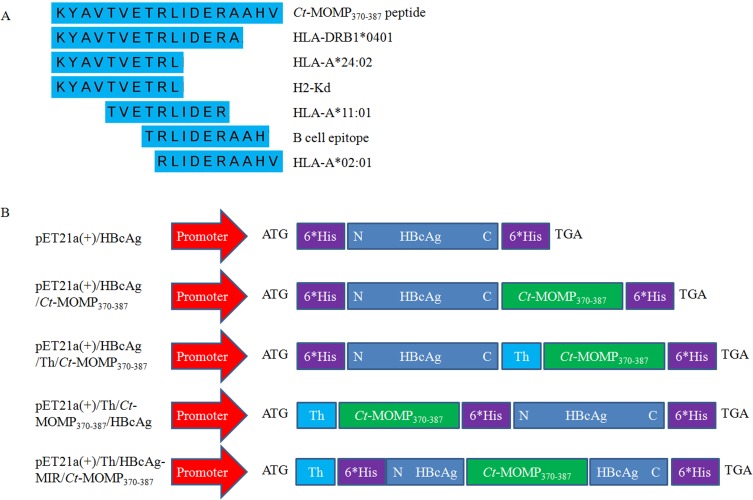
Prediction of *Ct* MOMPm and construction of three kinds of recombinant vectors **A.** Schematic representation of epitopes contained in the MOMP_370-387_ peptide. **B.** Schematic representation of three kinds of recombinant vectors. “N” or “C” means N-terminus or C-terminus of HBcAg sequence, respectively. “Th” means the universal helper T-lymphocyte epitope (PADRE). “6*His” means 6 His-tags.

Several peptides containing both HLA and H2 restricted cytotoxic T-lymphocyte (CTL)/Th epitopes were screened by analyzing the amino acid sequence of *Ct* MOMP, from which the peptide of MOMP_370-387_ containing the reported B-cell epitope (TRLIDERAAH) [13] was selected to be a candidate for MOMPm (Figure 1A).

**Figure 2:**
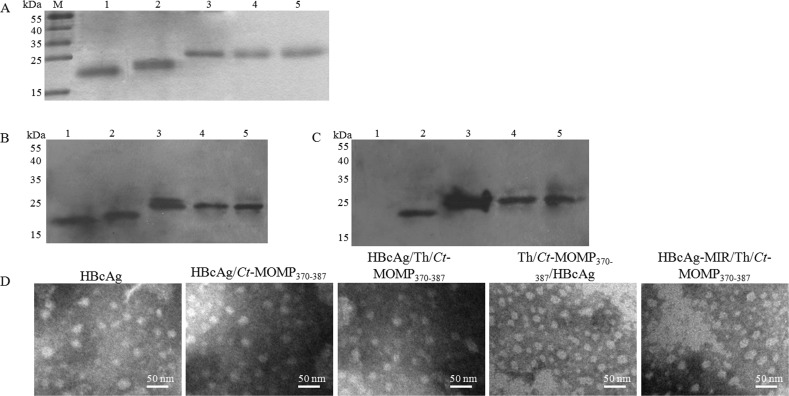
Expression of recombinant proteins and formation of HBcAg/MOMPm chimeric VLPs **A.** Comassie blue-stained SDS-PAGE gel of the purified recombinant proteins. **B.** Western blot analysis of recombinant proteins with anti-HBcAg antibody. **C.** Western blot analysis of recombinant proteins with anti-*Ct* antibody. In western blot analysis, SDS-PAGE gels were run under the same experimental conditions in section **A.** Then, proteins were transferred to a polyvinylidene fluoride membrane for western blot analysis with indicated antibodies. M, protein marker; 1, the purified recombinant HBcAg protein; 2, the purified recombinant HBcAg/*Ct*-MOMP_370-387_ protein; 3, the purified recombinant HBcAg/Th/*Ct*-MOMP_370-387_ protein; 4, the purified recombinant Th/*Ct*-MOMP_370-387_/HBcAg protein; 5, the purified recombinant HBcAg-MIR/Th/*Ct*-MOMP_370-387_ protein. **D.** The formation of HBcAg/MOMPm chimeric VLPs observed by negative-stain electron microscopic analysis.

The recombinant vectors, pET21a(+)/HBcAg, pET21a(+)/HBcAg/*Ct*-MOMP_370-387_, pET21a(+)/HBcAg/Th/*Ct*-MOMP_370-387_, pET21a(+)/Th/*Ct*-MOMP_370-387_/HBcAg and pET21a(+)/HBcAg-MIR/Th/*Ct*-MOMP_370-387_ were confirmed to be constructed successfully by indicated restriction endonuclease digestion and sequencing. The structure of these vectors was shown in Figure 1B. The corresponding recombinant proteins were expressed, purified and confirmed by SDS-PAGE and western blot analysis. As shown in Figure 2A, 2B and 2C, the proteins with expected weights were detected. The formation of VLPs was observed by negative-stain electron microscopic analysis (Figure 2D).

### Immunogenicity of HBcAg/MOMPm chimeric VLPs

**Figure 3:**
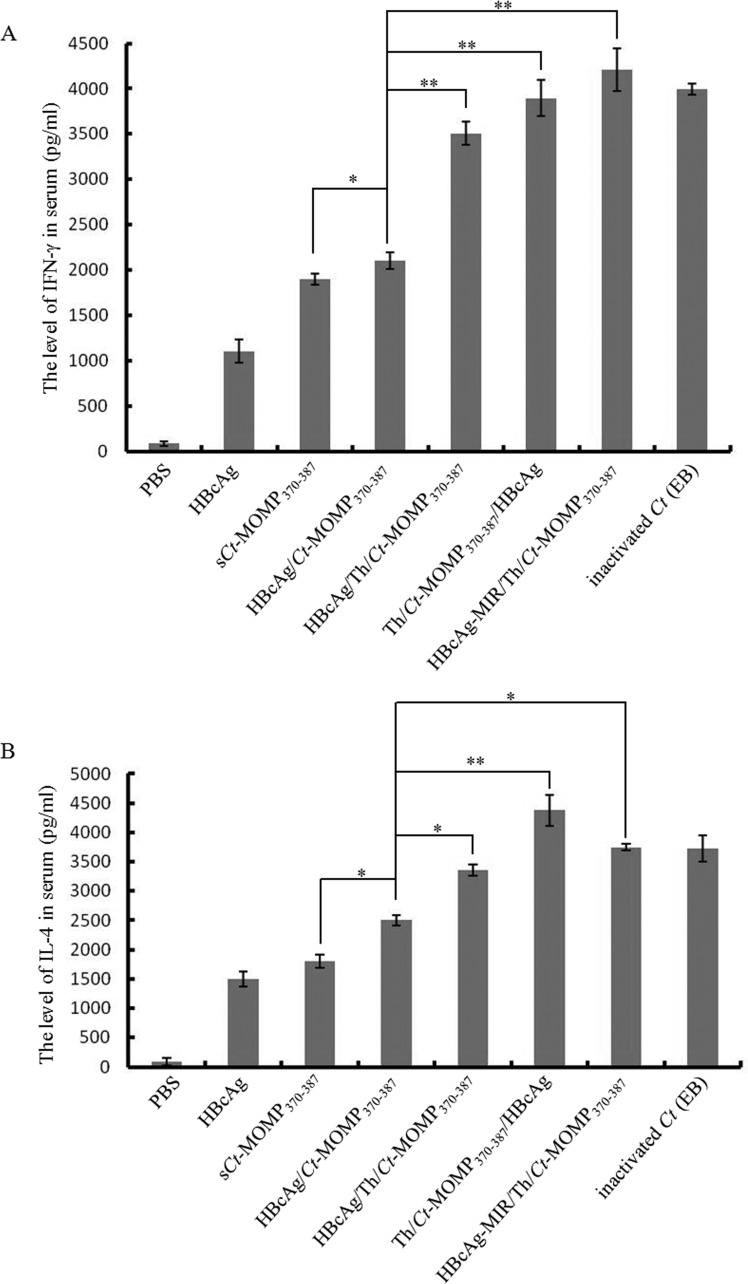
The levels of cytokines in the serum of mice immunized with different agents **A.** The level of IFN-γ in the serum. **B.** The level of IL-4 in the serum. All experiments were performed in triplicate and data are expressed as means ± SD (*n* = 3). Error bars represent standard deviations of replicate data points. *p < 0.05, **p < 0.01 between two indicated groups.

Serum cytokine concentrations were assayed at week 8. As shown in Figure 3A and 3B, compared with negative control groups (PBS and HBcAg), the secretion of the IFN-γ (produced by Th1 cells) and the IL-4 (produced by Th2 cells) in test groups was both significantly increased. Among the five test groups (synthetic *Ct*-MOMP_370-387_ peptide (s*Ct*-MOMP_370-387_), HBcAg/*Ct*-MOMP_370-387_, HBcAg/Th/*Ct*-MOMP_370-387_, Th/*Ct*-MOMP_370-387_/HBcAg, and HBcAg-MIR/Th/*Ct*-MOMP_370-387_), the secretion of IFN-γ and IL-4 in groups of HBcAg/*Ct*-MOMP_370-387_, HBcAg/Th/*Ct*-MOMP_370-387_, Th/*Ct*-MOMP_370-387_/HBcAg and HBcAg-MIR/Th/*Ct*-MOMP_370-387_ was significantly increased compared with the group of s*Ct*-MOMP_370-387_. Especially, the secretion of these two cytokines in groups of HBcAg/Th/*Ct*-MOMP_370-387_, Th/*Ct*-MOMP_370-387_/HBcAg and HBcAg-MIR/Th/*Ct*-MOMP_370-387_ was similar to that in positive control group (The inactivated *Ct* elementary body was called inactivated *Ct* (EB) for short.).

**Figure 4:**
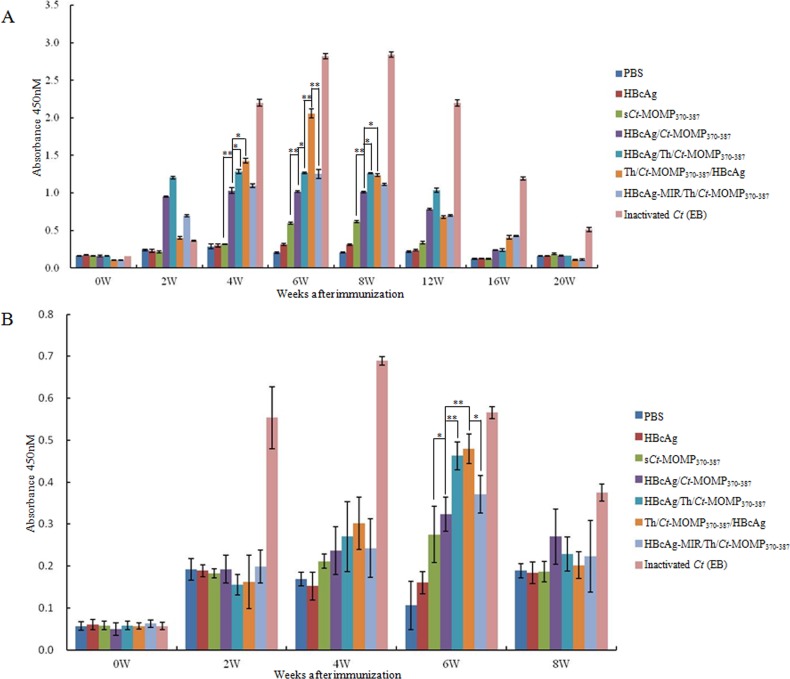
The levels of antibodies in the mice immunized with different agents The levels of *Ct*-specific serum IgG **A.** and secretory IgA **B.** were analyzed by ELISA. All experiments were performed in triplicate and data are expressed as means ± SD (*n* = 3). Error bars represent standard deviations of replicate data points. *p < 0.05, **p < 0.01 between two indicated groups.

Previous studies indicated that both IgG and secretory IgA could protect against genital chlamydial infection [14]. Therefore, the level of these antibodies in the serum and vaginal secretions of immunized mice was determined by ELISA. Except negative control groups, the level of *Ct*-specific serum IgG in test groups and positive control group rose gradually after immunization and reached the highest point during week 6 to 8. Among these five test groups, immunization with HBcAg/Th/*Ct*-MOMP_370-387_ induced higher level of IgG than the HBcAg/*Ct*-MOMP_370-387_ group. Furthermore, immunization with Th/*Ct*-MOMP_370-387_/HBcAg induced the highest level of IgG, which was significantly higher than that induced by HBcAg/Th/*Ct*-MOMP_370-387_ and HBcAg-MIR/Th/*Ct*-MOMP_370-387_ (Figure 4A).

Except negative control groups, the level of *Ct*-specific sIgA in vaginal secretions in test groups and positive control group also rose gradually after immunization. The level of sIgA in all of the test groups reached the highest point at week 6 and then decreased at week 8. Among the test groups, at week 6, immunization with Th/*Ct*-MOMP_370-387_/HBcAg induced the highest level of sIgA, followed by HBcAg/Th/*Ct*-MOMP_370-387_. Furthermore, the level of sIgA in these two groups was both significantly higher than that in the group of HBcAg/*Ct*-MOMP_370-387_. The level of sIgA in the group of HBcAg/*Ct*-MOMP_370-387_ was significantly higher than that in the group of s*Ct*-MOMP_370-387_ (Figure 4B).

**Figure 5:**
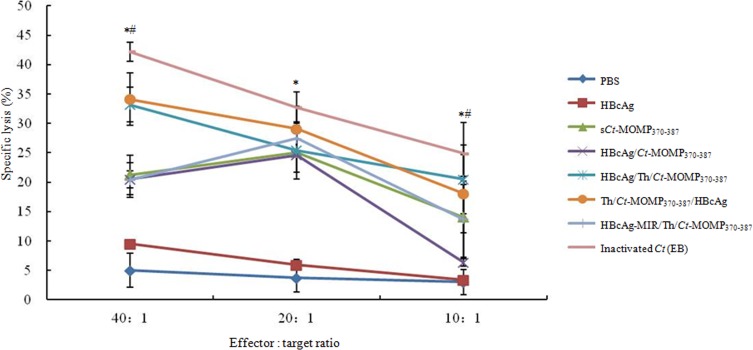
The CTL response of splenic lymphocytes against *Ct* in mice immunized with different agents was analyzed by a standard LDH assay Splenic lymphocytes derived from mice of test groups (s*Ct*-MOMP_370-387_, HBcAg/*Ct*-MOMP_370-387_, HBcAg/Th/*Ct*-MOMP_370-387_, Th/*Ct*-MOMP_370-387_/HBcAg, and HBcAg-MIR/Th/*Ct*-MOMP_370-387_) exhibited significantly higher cytotoxicity than those from negative control groups (PBS and HBcAg) (*p < 0.05) at any of effector/target cell ratio. Splenic lymphocytes from the group of Th/*Ct*-MOMP_370-387_/HBcAg exhibited the highest cytotoxicity at the effector/target ratio of 20:1 and 40:1 (#*p* < 0.05). All experiments were performed in triplicate and data are expressed as means ± SD (*n* = 3). Error bars represent standard deviations of replicate data points.

The cytotoxicity of splenic lymphocytes from the immunized mice was further analyzed by CTL assay. As shown in Figure 5, at any of effector/target cell (E/T) ratio, splenic lymphocytes derived from mice of test and positive groups exhibited significantly higher cytotoxicity than negative control groups (PBS and HBcAg). Meanwhile, splenic lymphocytes from the group of Th/*Ct*-MOMP_370-387_/HBcAg exhibited the highest cytotoxicity at the effector/target ratio of 20:1 and 40:1.

### Protective immunity induced by HBcAg/MOMPm chimeric VLPs

**Figure 6:**
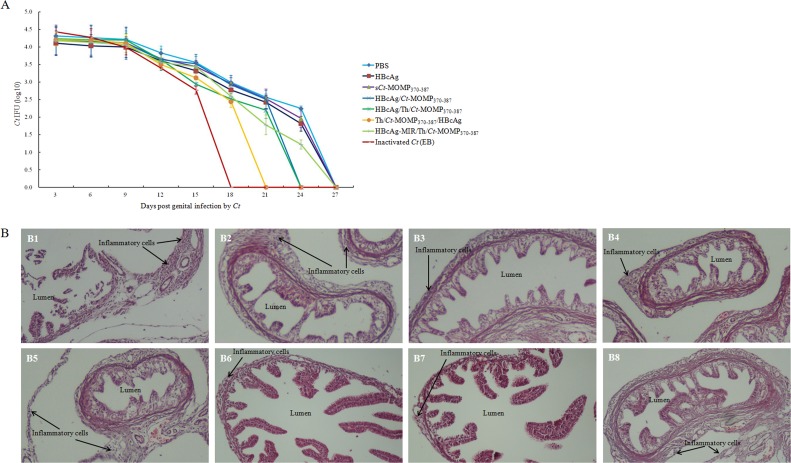
Immunization with HBcAg/MOMPm chimeric VLPs protects mice against *Ct* infection **A.** *Ct* shedding (IFUs) was measured from vaginal swabs every 3 days post challenge. The mice in five test groups were highly resistant to infection (as indicated by the lower IFU number and the shorter time to resolution of the infection) compared with mice in negative control groups. **B.** The effect of immunization with HBcAg/MOMPm chimeric VLPs on inflammatory pathologies in mouse upper genital tract. Thirty days after chlamydial infection, mice were sacrificed for pathology observation. The genital tract tissues were sectioned for microscopic observation of inflammatory pathologies. Representative H&E stained section images from each group of mice genital tracts tissue (original magnification, ×100) were shown. The location of lumen and examples of inflammatory cell infiltration were indicated. B1, PBS; B2, HBcAg; B3, s*Ct*-MOMP_370-387_; B4, HBcAg/*Ct*-MOMP_370-387_; B5, HBcAg/Th/*Ct*-MOMP_370-387_; B6, Th/*Ct*-MOMP_370-387_/HBcAg; B7, HBcAg-MIR/Th/*Ct*-MOMP_370-387_; B8, inactivated *Ct* (EB). Note that immunization with HBcAg/MOMPm chimeric VLPs significantly reduced both lumen dilatation and inflammatory cell infiltration.

Protective efficacy was evaluated by challenging the mice with 10^6^ inclusion-forming units (IFUs) *Ct* via the intra-vaginal route at week 8. Protection was assessed by isolation of *Ct* from vaginal swabs and comparing the number of IFUs recovered from immunized mice at the indicated time points. As shown in Figure 6A, compared with mice in negative control groups, mice in five test groups were highly resistant to infection (as indicated by the lower IFU number and the shorter time to resolution of the infection). Except the positive control group, the most significant level of protection was observed in mice immunized with Th/*Ct*-MOMP_370-387_/HBcAg, followed by mice immunized with HBcAg/Th/*Ct*-MOMP_370-387_ and HBcAg/*Ct*-MOMP_370-387_, respectively. All mice immunized with HBcAg/MOMPm chimeric VLPs showed complete resolution of the infection within 24 days after the initial challenge except those immunized with HBcAg-MIR/Th/*Ct*-MOMP_370-387_. These results indicate that HBcAg as a carrier for *Ct* MOMPm peptide enhances protection against genital chlamydial infection.

We further evaluated the effect of immunization with HBcAg/MOMPm chimeric VLPs on inflammatory pathologies in mouse upper genital tract. Thirty days after the intra-vaginal challenge infection, mice were sacrificed and mouse genital tract tissues were collected for pathology evaluation. As shown in the results of histology (Figure 6B), the mice immunized with inactivated *Ct* (EB) showed nearly normal oviduct lumens and only scattered inflammatory cells in oviduct tissues. The mice immunized with HBcAg/MOMPm chimeric VLPs exhibited milder luminal dilatation and inflammatory cell infiltration. However, highly dilated oviduct lumens and extensive inflammatory infiltration were observed in the oviduct tissues of mice immunized with s*Ct*-MOMP_370-387_, HBcAg or PBS. Furthermore, some of the fallopian tubal folds were even disappeared in the tissues of negative control groups.

The above results demonstrated that the immunity induced by MOMPm could shorten the time course of live organism shedding and reduce the inflammatory damages in the mouse oviducts. Therefore, a chimeric vaccine based on the HBcAg platform has significant advantages in protecting vaccinated recipients from chlamydial infection.

## DISCUSSION

Currently, selection of appropriate target antigens is the main problem in the development of *Ct* vaccine [15]. Although killed vaccines offer some degree of protection, they can lead to detrimental side effects [16]. MOMP is crucial to chlamydial infection and pathogenesis due to its interaction with host proteins and inhibition of phagosome-lysosome fusion [17]. Owing to its immunogenicity, MOMP has been widely used as a candidate antigen to develop a vaccine against chlamydial infection [18-20]. However, it is difficult to obtain recombinant MOMP in a native form with intact, conformationally relevant epitopes due to its cysteine-rich structure. Therefore, MOMPm epitopes (T- and B-cell epitope-rich peptide clusters) have recently been designed as the immunogen in order to induce a cellular and humoral immune synergetic effect [21, 22].

Multi-epitope vaccine could not only solve problems of immunodominance but also generate broad immune response [23, 24]. However, multi-epitope vaccine needs a suitable antigen delivery and release vehicle to stimulate effective immune response. Several carrier systems have been used to develop Ct vaccines such as viral or bacterial ghosts, cytokine fusion constructs, CpG lipophilic ISCOMs, dendritic cell, and VLPs [22, 25-28].

VLPs preserve the native antigenic conformation of the immunogenic proteins without any risks of infection and thus offer unique advantages in terms of safety and immunogenicity over other vaccine carrier systems [29, 30]. As reported in our previous studies, using hepatitis B virus surface antigen and human papillomavirus major capsid protein L1 as delivery vectors for *Ct* MOMPm DNA vaccines could induce protective immunity against *Ct* infection [21, 22]. However, DNA vaccines still have some disadvantages, such as their weak immunogenicity and the lack of efficient DNA delivery platforms (reviewed in [31, 32]). HBcAg that has been used as vaccine carrier systems for almost three decades holds a unique position among VLP carriers because of its high-level expression and efficient particle formation in virtually all known expression systems [8, 9]. Therefore, in this study, HBcAg was used as a delivery system for *Ct* MOMPm. We analyzed the efficacy of the novel HBcAg/MOMPm chimeric vaccine. The results of SDS-PAGE and western blot analysis showed that the HBcAg fused proteins were effectively expressed and these proteins also formed VLPs successfully as expected (Figure 2).

The *Ct* MOMPm designed in this study not only contains HLA-A*02:01, HLA-A*11:01, HLA-DRB1*0401, and HLA-A*24:02 restricted T-cell epitopes and a reported B-cell epitope but also contains mouse H2-Kd restricted CTL epitopes (Figure 1) in order to evaluate the immune response on murine model.

Lymphocyte homeostasis is required to maintain the normal immune function [33]. CD4^+^ T-cells, an important kind of lymphocytes, can be divided into Th1 and Th2 cells according to different cytokine secretion. Th1 cells mainly produce IFN-γ and IL-2, which are critical to cellular immunity and CTL responses, whereas Th2 cells mainly produce IL-4 and IL-10, which enhance antibody production by B cells and improve humoral immunity [34]. The result of our cytokine assay showed that immunization with HBcAg/*Ct*-MOMP_370-387_ and HBcAg/Th/*Ct*-MOMP_370-387_ both resulted in significantly higher level expression of IFN-γ and IL-4 compared with the group of s*Ct*-MOMP_370-387_ and negative groups (Figure 3), according to which we speculated that immunization with HBcAg/*Ct*-MOMP_370-387_ and HBcAg/Th/*Ct*-MOMP_370-387_ could induce both humoral and cellular immune responses. Interestingly, this hypothesis was confirmed by detection of *Ct*-specific serum IgG and secretory IgA and CTL assay. As shown in Figure 4A and 4B, higher levels of IgG and sIgA were induced in groups of HBcAg/*Ct*-MOMP_370-387_ and HBcAg/Th/*Ct*-MOMP_370-387_ than that in group of s*Ct*-MOMP_370-387_. Similar results were obtained in CTL assay (Figure 5). These results indicate that HBcAg is also an excellent carrier for development of the *Ct* MOMPm vaccine and that Th epitope can enhance the immunogenicity of the *Ct* MOMPm. In addition, as shown in Figure 3, Figure 4 and Figure 5, the N-terminus fusion (Th/*Ct*-MOMP_370-387_/HBcAg) exhibited the best immunogenicity in the three ways that *Ct* MOMPm was fused with HBcAg, which was inconsistent with previous studies [9, 35]. In those studies, the MIR of HBcAg is the preferable insertion site for foreign proteins. There may be at least two reasons for the difference between our results and previous studies. The first one is the different lengths of foreign proteins and the second one is the different insertion sites of Th epitope. Further studies need to be done to characterize the mechanism of the best immunogenicity by N-terminus fusion of foreign proteins to HBcAg in our results.

We also investigated the ability of the HBcAg/MOMPm chimeric VLPs to induce protective immunity. As shown in Figure 6A and 6B, mice immunized with the HBcAg/*Ct*-MOMP_370-387_ and HBcAg/Th/*Ct*-MOMP_370-387_ both induced protection against genital chlamydial infection. Furthermore, immunization with HBcAg/Th/*Ct*-MOMP_370-387_ induced more significant level of protection. These results indicate that HBcAg as a carrier for *Ct* MOMPm peptide significantly enhances protection against genital chlamydial infection and that the Th epitope also enhances this protection. Additionally, MOMPm fused to different insertion sites (C-terminus, N-terminus or MIR) of HBcAg exhibited different levels of protection against *Ct* challenging and the best way was N-terminus fusion, which was generally consistent with the results of immunogenicity assay.

Taken together, our results support the notion that vaccines based on chimeric HBcAg carrying *Ct* MOMPm could be an effective immune prophylactic for chlamydial infection in murine model system and that different ways of *Ct* MOMPm fused with HBcAg could induce different levels of protective immune response against genital chlamydial infection. Therefore, our results could be useful for the development of vaccines to protect human beings against chlamydial infection.

## MATERIALS AND METHODS

### Ethics statement

Our study had been approved by Animal Care and Use Committee of Zhejiang Province, China. All animal procedures were performed according to guidelines developed by the China Council on Animal Care and protocol approved by Animal Care and Use Committee of Zhejiang Province, China.

### Chlamydial organisms and reagents

*Ct* serovar E organisms (ATCC VR-348B) were grown, purified, and titrated as previously described [36]. Samples with chlamydial infection in various assays were prepared as previously described [21]. Polyclonal rabbit IgG antibody against *Ct* were prepared by immunizing rabbits with inactivated *Ct* (EB) in our laboratory. Mouse monoclonal antibody against HBcAg was purchased from Abcam^®^. Horseradish peroxidase (HRP)-labeled goat anti-rabbit and goat anti-mouse secondary antibodies were also purchased from Abcam^®^.

### Screening and generating Ct MOMPm

Multi-epitope peptide was screened as following. Briefly, the amino acid sequence of MOMP from *Ct* serovar E (http://www.uniprot.org/uniprot/P17451) was obtained from the Swiss-prot database (http://www.expasy.org/sprot/), which was analyzed using SYFPEITHI program (http://www.syfpeithi.de/bin/MHCServer.dll/EpitopePrediction.htm) to predict both HLA and H2 restricted CTL epitopes. The peptide (MOMP_370-387_) with high score containing the reported B-cell epitope (TRLIDERAAH) [13] was selected to be a candidate for MOMPm, according to which the nucleic acids of MOMPm were synthesized.

### Constructing the HBcAg/MOMPm plasmid

HBcAg gene was amplified by polymerase chain reaction (PCR) from the serum of HBV carriers with standard protocols using the following primer pair: forward, 5′-ATGCAACTTTTTCACCTCTGCCTAAT-3′ and reverse, 5′-CTAACATTGAGATTCCCGAGATTGAGA-3′. The amino acid sequence encoded by our HBcAg gene was consisted with that retrieved in NCBI (GenBank ID: X01587). Then, HBcAg gene with 6 His-tags at the N-terminus was amplified using the following primer pair: forward, 5′ - GGAATTCCATATGCAC CATCATCATCATCATGACATTGACCCGTATAAAG - 3′ and reverse, 5′-GGGGTACCACATTGAGATTCCCG-3′ and subcloned into the pET32a(+) vector between restriction endonucleases sites of *Nde* I and *Kpn* I (named as pET32a(+)/HBcAg). The pET32a(+)/HBcAg vector was digested by double restriction endonucleases at the sites of *Nde* I *and Eco*RI and the shorter nucleic acids was subcloned into the corresponding sites of pET21a(+) vector (named as pET21a(+)/HBcAg). The nucleic acids of MOMPm with *Kpn*I and *Bam*HI *r*estriction endonucleases sites were synthesized (forward, 5′ - CAAATACGCTGTTACCGTTGAAA CCCGTCTGATCGACG AACGTGCTGCTCACGTTG - 3′ and reverse, 5′ - GATCCAACGTGAGCAGCACGTTCGTCGATCAG ACGGGTTTCAA CGGTAACAGCGTATTTGGTAC - 3′) and subcloned into the corresponding sites of pET21a(+)/HBcAg vector (named as pET21a(+)/HBcAg/Ct-MOMP_370-387_). To enhance the immunogenicity of MOMPm, the nucleic acids of universal helper T-lymphocyte epitope (Th epitope, PADRE) [37-39] with *Kpn*I and *Bam*HI *r*estriction endonucleases sites (forward, 5′ - CGCTAAAT TCGTTGCTGCTTGGACCC TGAAAGCTGCTGCTG - 3′ and reverse, 5′ - GATCCAGCAGCAGCTTT CAGGGTCCAAGCAGCAACGAATTTAGCGGTAC - 3′) and the nucleic acids of mMOMP_370-387_ epitope with *Bam*HI and *Hind*III *r*estriction endonucleases sites (forward, 5′- GATCCAAATACGCTGTTACCGTTGAAACCCGT CTGATCGACGAACGTGCTGCTCACGTTA - 3′ and reverse, 5′- AGCTTCTAAACGTGAGCAGCACGTTCGTCGATCA GACGGGTTTCAACGGTAACAGCGTATTTG - 3′) were respectively synthesized and subcloned into the pET21a(+)/HBcAg vector between restriction endonucleases sites *Kpn*I and *Hind*III (named as pET21a(+)/HBcAg/Th/Ct-MOMP_370-387_). The nucleic acids of Th epitope and mMOMP_370-387_ epitope with two *Nde*I *r*estriction endonucleases sites (forward, 5′ - TATGGCTAAATTCGTTGCTGCTTGGACCCTGAAA GCTGCTGCTGGATCCAAATACGCTG TTACCGTTGAAACCCGTCTGA TCGACGAACGTGCTGCTCACG TTCA - 3′ and reverse, 5′ - TATGAA CGTGAGC AGCACG TTCGTCGATCAGACGGGTTT CAACGGTAACAGCG TATTT GGATCCAGCAGCAGCT TTCA GGGTCCAA GCAGCAACGAATTTAGCCA - 3′) were synthesized and subcloned into the corresponding sites of pET21a(+)/HBcAg vector (named as pET21a(+)/Th/Ct-MOMP_370-387_/HBcAg). The nucleic acids of mutant HBcAg gene (restriction endonuclease sites of *Bam*HI and *Sac*I were generated by synonymous mutation at amino acid site of 74 and 82 because MIR was located in this region) with Th epitope were synthesized and subcloned into the pET21a(+) vector between restriction endonucleases sites *Nde*I and *Hind*III (named as pET21a(+)/HBcAg-MIR). Then, the nucleic acids of mMOMP_370-387_ epitope with *Bam*HI and *Sac*I *r*estriction endonucleases sites (forward, 5′-GATCCAAATACGCTGTTACCGTTGAAACCCG TCTGAT CGACGAACGTGCTGCTCACGTTGAGCT - 3′ and reverse, 5′-CAACGTGAGCAGCACGTTCGTCGA TCA GACGGGTTTCAACGGTAACAGCGTATTTG - 3′) were synthesized and subcloned into the corresponding sites of pET21a(+)/HBcAg-MIR vector (named as pET21a(+)/HBcAg-MIR/Th/Ct-MOMP_370-387_). All of the recombinant vectors were confirmed by restriction endonuclease digestion and sequencing.

### Preparing HBcAg/MOMPm chimeric VLPs

E. coli Rosseta (DE3) was transformed with three kinds of recombinant vectors respectively and induced by IPTG (0.8mmol/L) to express corresponding recombinant proteins with 6 His-tags including HBcAg, HBcAg/*Ct*-MOMP_370-387_, HBcAg/Th/*Ct*-MOMP_370-387_, Th/*Ct*-MOMP_370-387_/HBcAg and HBcAg-MIR/Th/*Ct*-MOMP_370-387_. Purification of HBcAg VLPs and HBcAg/MOMPm chimeric VLPs was performed as described in previous studies with minor modifications [40, 41]. Briefly, the recombinant proteins were adjusted to dissociation buffer (6 M urea, 10 mM imidazole, 50 mM sodium phosphate buffer, pH 8.0, and 50 mM NaCl), and incubated at 37°C for 30 min; all subsequent steps were performed at room temperature. The solutions were mixed at the desired molar ratios, and bound to a Ni^2+^-nitrilotriacetic acid (Ni^2+^-NTA) column pre-equilibrated in dissociation buffer (usually 1-2 ml protein solution for a 2 ml gel bed column). The urea concentration was decreased by washing with 2 ml each of washing buffer (20 mM imidazole, 50 mM sodium phosphate, pH 8.0, and 50 mM NaCl) containing 5, 4, 3, 2, 1 and 0.5 M urea, followed by pure washing buffer, then washing buffer containing 300 mM NaCl. Finally, twice the gel bed volume of elution buffer (250 mM imidazole, 50 mM sodium phosphate buffer, pH 8.0, and 300 mM NaCl) was added. After 1 h incubation, the proteins were eluted by gravity flow. To separate CLPs from non-assembled forms, dialyzed and concentrated samples were subjected to sedimentation in 10-60% (wt/vol) sucrose density gradient ultracentrifugation. The purified proteins were confirmed by western blot analysis and the reassembled VLPs were observed by negative-stain electron microscopic analysis.

### Western blot

The recombinant proteins were separated by SDS-PAGE and then transferred to a polyvinylidene fluoride membrane (Millipore, Billerica, MA, USA). The membrane was blocked with blocking buffer (5 % skimmed milk in Tris-HCl buffered saline) and then incubated with indicated primary antibodies over night at 4°C, followed by HRP-conjugated secondary antibodies at room temperature for 1 h. The protein bands were visualized by Luminata Classico Western HRP Substrate as per the manufacturer's instructions (Millipore, Billerica, MA, USA).

### Negative-stain transmission electron microscopy

Particle preparations were spotted onto carbon-coated copper grids and negatively stained with 3% (w/v) phosphotungstic acid. The grids were examined using an FEI Tecnai 20 transmission electron microscope operating at 200 kV.

### Analyzing the immunogenicity of HBcAg/MOMPm chimeric VLPs

A total of 40 BALB/c female mice (H2-Kd) at the age of 6 to 8 weeks old were purchased from Shanghai Experimental Animal Center at the Chinese Academy of Sciences and randomly divided into 8 groups. The mice of each group were randomly assigned to receive immunization with PBS, HBcAg, s*Ct*-MOMP_370-387_, HBcAg/*Ct*-MOMP_370-387_, HBcAg/Th/*Ct*-MOMP_370-387_, Th/*Ct*-MOMP_370-387_/HBcAg, HBcAg-MIR/Th/*Ct*-MOMP_370-387_ and inactivated *Ct* (EB). Each mouse was immunized with 100 μl indicated samples (dissolved in PBS, 1 mg/ml) by subcutaneous injection at the first day of week 0, 2, 4. The tail blood was respectively collected at the last day of week 0, 2, 4, 6, 8, 10, 12, 14, 16, 18, and 20 and the vaginal washes were respectively collected at the last day of week 0, 2, 4, 6, and 8. Then, indirect ELISA was performed to detect *Ct*-specific serum IgG and secretory IgA. Another 40 mice were also divided into 8 groups and immunized as mentioned above to perform CTL assay. The mouse splenic lymphocytes were isolated for CTL assay at the last day of week 7.

### Determination of *Ct*-specific serum IgG and secretory IgA

Microplates were coated with purified whole *Ct* protein (BestBio biotechnology co. Shanghai, China). After incubation at 4°C overnight, the plates were rinsed with PBS solution containing 0.05 % Tween 20 (PBST). The coated plates were blocked with 100 μl blocking buffer (5 % skimmed milk in PBST) at 37°C for 1 h. After being rinsed for 5 times with PBST, the plates were incubated with immune sera (diluted at 1:1000 in blocking buffer, 100 μl/well) or vaginal washes (diluted at 1:10 in blocking buffer, 100 μl/well) at 37°C for 2 h. After being rinsed for 5 times with PBST, the plates were incubated with HRP-conjugated goat anti-mouse IgG antibody (diluted at 1:1000 in blocking buffer, 100 μl/well) or HRP-conjugated goat anti-mouse sIgA antibody (diluted at 1:2000 in blocking buffer, 100 μl/well) at 37°C for 1 h. After being rinsed again, the plates were incubated with 3, 3′, 5, 5′-tetramethylbenzidine (TMB)-H_2_O_2_ solution (100 μl/well) at room temperature for 20 min and then with 2 mol/L H_2_SO_4_ (50 μl/well) to stop the reaction. The resultant color was measured at 450 nm in a Bio-tek ELISA microplate reader.

### Cytokine assay

The levels of interferon (IFN)-γ (a classical Th1-derived cytokine) and interleukin (IL)-4 (a classical Th2-derived cytokine) in the serum of mice from different groups at week 8 were measured using commercially available ELISA cytokine kits (eBioscience, USA) as per the manufacturer's protocol.

### CTL assay

Short-term CTL cultures were generated as previously described with some modifications [42, 43]. Briefly, at week 7, splenic lymphocytes were collected from immunized mice as the effector cells, 5×10^6^ of which were co-cultured with 2×10^5^ inactivated *Ct* (EB) in presence of 0.5 ng/ml IL-7 and 20U/ml IL-2 for 5 days in 24-well tissue culture plates. The cytolytic activity of the cultures was determined using a lactate dehydrogenase (LDH) release assay kit (Promega, Wisconsin, USA). The target cells, Chlamydia-infected BALB/3T3 cells, were dispensed into 96-well round-bottom plates (10^4^ cells/well) and then serial dilutions of effector cells were added to the wells. The plates were centrifuged at 1500 rpm for 10min. After 4 h, the plates were centrifuged again. 50 μl supernatant was transferred to another plate and mixed with the substrate mix provided in the test kits. The reaction was stopped after 30 min and read using a Bio-tek ELISA microplate reader at a wave-length of 490 nm. Specific lysis was determined as follows: % specific lysis = 100 × [(release in the presence of CTL - spontaneous release) / (maximal release spontaneous release)]. In all experiments, the spontaneous release was less than 30 % of the maximal release.

### Evaluating the protective effect of HBcAg/MOMPm chimeric VLPs against genital chlamydial infection in mice

Eight groups of mice (5 mice/group) were immunized as described above. At week 8, the mice were challenged intra-vaginally with 10^6^ IFUs of live *Ct* serovar E. Five days prior to infection, each mouse was injected with 2.5 mg Depo-provera (Pharmacia Upjohn, Kalamazoo, MI, USA) subcutaneously to synchronize menstrual cycle and increase mouse susceptibility to chlamydial infection. The protective effect was assessed by calculating the isolation of Chlamydia from vaginal swabs. Vaginal swabs were obtained once every 3 days after the intra-vaginal infection until two consecutive negative detection results were obtained from the same mouse. Thirty days after chlamydial infection, the reproductive tract was dissected and histopathological comparisons among mice from the 8 groups were then performed. Both the inflammatory cell infiltration and luminal dilatation were evaluated by double-blind examiner.

### Statistical analysis

ANOVA test was performed to analyze data from multiple groups and a two-tailed Student *t* test to compare the means between two groups. A chi-squared test was used for comparing the rate of incidence between two groups. The level of significance was set at *p* < 0.05.
